# Resistance gene enrichment sequencing refines the *Brassica napus* NLRome

**DOI:** 10.1093/plphys/kiae631

**Published:** 2024-11-28

**Authors:** Jiaxu Wu (吴家旭), Soham Mukhopadhyay, Edel Pérez-López

**Affiliations:** Départment de Phytologie, Faculté des Sciences de l'agriculture et de l'alimentation, Université Laval, Québec City, QC, Canada G1V 0A6; Centre de Recherche et d’innovation sur les Végétaux (CRIV), Université Laval, Québec City, QC, Canada G1V 0A6; Institute de Biologie Intégrative et des Systèmes (IBIS), Université Laval, Québec City, QC, Canada G1V 0A6; L’Institute EDS, Université Laval, Québec City, QC, Canada G1V 0A6; Départment de Phytologie, Faculté des Sciences de l'agriculture et de l'alimentation, Université Laval, Québec City, QC, Canada G1V 0A6; Centre de Recherche et d’innovation sur les Végétaux (CRIV), Université Laval, Québec City, QC, Canada G1V 0A6; Institute de Biologie Intégrative et des Systèmes (IBIS), Université Laval, Québec City, QC, Canada G1V 0A6; L’Institute EDS, Université Laval, Québec City, QC, Canada G1V 0A6; Départment de Phytologie, Faculté des Sciences de l'agriculture et de l'alimentation, Université Laval, Québec City, QC, Canada G1V 0A6; Centre de Recherche et d’innovation sur les Végétaux (CRIV), Université Laval, Québec City, QC, Canada G1V 0A6; Institute de Biologie Intégrative et des Systèmes (IBIS), Université Laval, Québec City, QC, Canada G1V 0A6; L’Institute EDS, Université Laval, Québec City, QC, Canada G1V 0A6

## Abstract

Resistance gene enrichment sequencing produces a complete repertoire of nucleotide-binding leucine-rich repeat receptors for Brassica napus, overcoming prior limitations in gene annotation.

Dear Editor,

Canola (*Brassica napus* L.) is primarily cultivated as an oilseed crop with substantial economic value (Javed et al. 2023). However, the prevalence of diseases such as clubroot, sclerotinia stem rot, and blackleg threatens the canola industry worldwide (Chen et al. 2021). To improve disease resistance, it is crucial to accurately annotate nucleotide-binding leucine-rich repeat receptors (NLRs), key components of plant immune systems (Contreras et al. 2023). This study focuses on refining the NLR repertoire of the “Westar” canola cultivar (hereafter Westar canola), which is widely used in genetic mapping, transformation studies and pathogenicity assays (Yang et al. 2023; Zou and Fernando 2024).

A long-read-based high-quality genome was released for Westar canola, which is predicted to carry 97,514 annotated genes (Song et al. 2020; Yang et al. 2023). While studying the NLR repertory of Westar canola based on the available gene annotation, we found the presence of only 345 genes with domain signatures associated with NLR proteins using InterproScan and NLRtracker (Supplementary Table S1; Jones et al. 2014; Kourelis et al. 2021). This number is very low compared to other *B. napus* canola cultivars such as “ZS11' (Genome Warehouse: GWHANRE00000000) and “Darmor” (NCBI RefSeq: GCF_020379485.1) which are predicted to carry 597 and 641 NLR loci, respectively (Alamery et al. 2018; Chen et al. 2021). This made us wonder if (i) the Westar genome was properly annotated, and if (ii) we could further refine the canola NLRome. To answer those questions, we produced the first canola NLRome using the Westar genome and resistance gene enrichment sequencing (RenSeq), a method that has advanced resistance (R) genes discovery and cloning (Jupe et al. 2013; Thomas et al. 2024). RenSeq is a targeted sequencing method offering a less expensive and less laborious alternative for NLR genetic mapping or NLR discovery studies (Jupe et al. 2013).

In this study, a set of 74,738 RenSeq baits was designed based on the “ZS11” canola genome NLR nucleotide sequences by Arbor Bioscience (Fig. 1A; Supplementary Fig. S1 and File S1). We used 80 nt probes and 3× tilling with a total target size of 2,951,019 nt and an average GC content of 37.6%. Targets were softmasked for repeats against the eudicot database. Strings of Ns 1-10 nt long were replaced with Ts. High molecular weight genomic DNA was extracted from 3-week-old homozygous doubled-haploid Westar canola seedlings with a CTAB method as previously described (Jupe et al. 2013). Illumina library enrichment and NLR capture sequencing were performed by myBaits Custom Hybridization Capture Kits (Daicel Arbor Biosciences, USA) following their standard protocols, resulting in 45 million 150 bp paired-end Illumina reads (Bioproject: PRJNA1137270). Next, we mapped the reads to the Westar genome using BWA-MEM2 (Vasimuddin et al. 2019) and visualized them using Geneious Prime (https://www.geneious.com/). In parallel, the NLR-Annotator (Steuernagel et al. 2020) was used to predict NLR loci across the genome.

**Figure 1.**
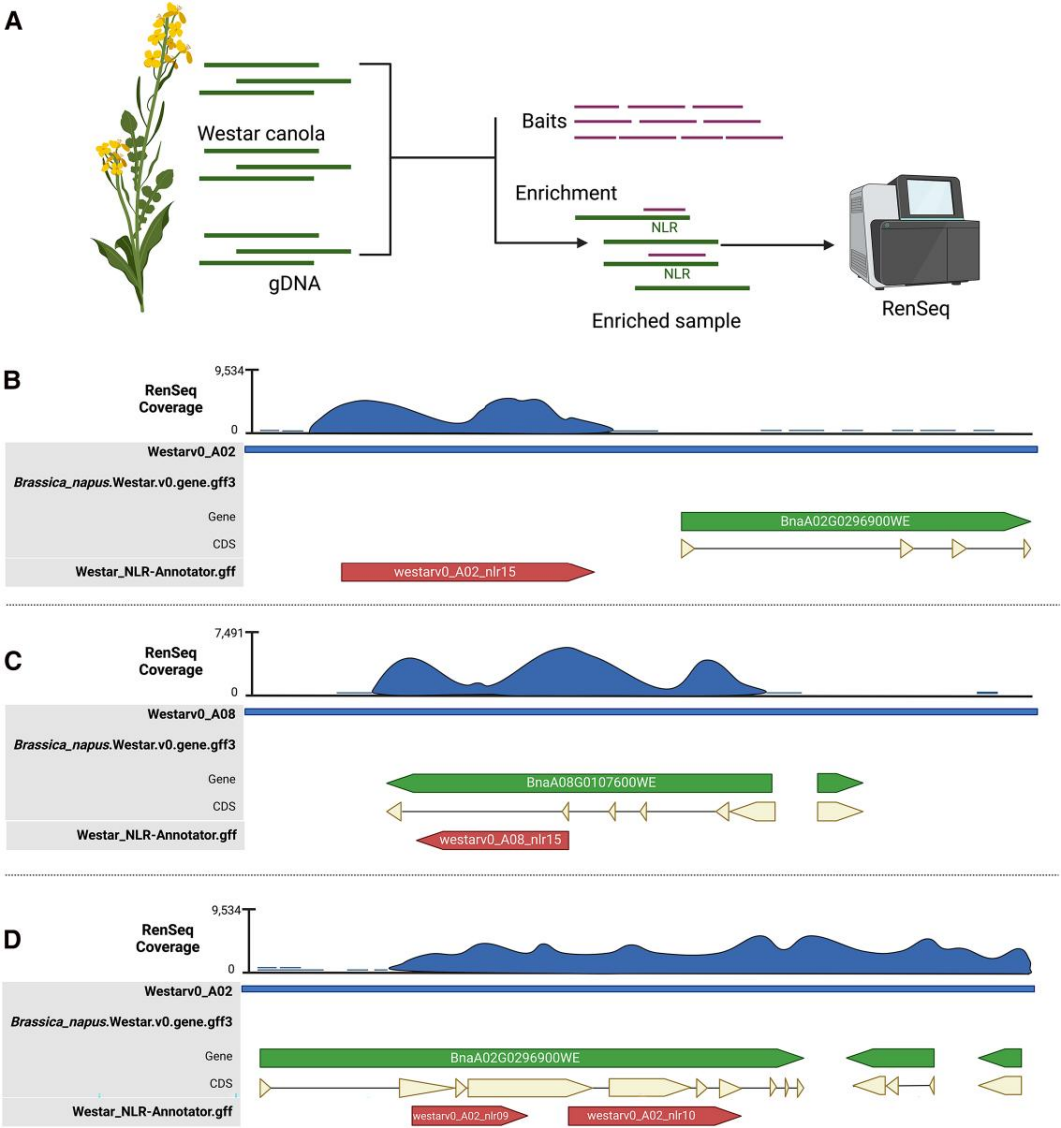
Overview of *Brassica napus* NLRome refinement using RenSeq and examples of wrongly annotated NLRs in the Westar canola genome. **A)** Schematic representation of the RenSeq workflow followed in this study. Genomic DNA (sDNA) from Westar canola was enriched for NLRs using a customized NLR RNA bait-library. The enriched sample was sequenced using the Illumina NovaSeq 6000 platform. Scheme created using Biorender. **B)** Example of NLR on chromosome A02 missed in reference annotation file with high RenSeq coverage and predicted by NLR-Annotator. **C)** A clubroot resistance gene model, *Crr1a* allele on chromosome A08 is wrongly annotated in the reference annotation file with high RenSeq coverage and predicted by NLR-Annotator. **D)** Example of two NLRs annotated as a unique NLR on chromosome A02 in the reference annotation file. In panels B to D, CDS stands for coding sequence. Blue indicates RenSeq coverage, green represents the originally annotated gene, beige denotes the CDS, and red highlights the newly reannotated NLRs.

Using Geneious, we overlaid the NLR-Annotator prediction and the gene annotation supplied with the genome (Supplementary File S2) on top of the BWA-MEM2 mapping. This allowed us to manually inspect the genes that are correctly annotated with sufficient RenSeq read depth (at least 50×) and NLR-Annotator coverage. All 345 genes that were previously identified by InterProScan and NLRtracker and annotated in the Westar genome were also identified using this methodology (Supplementary Fig. S2, Tables S1, and File S2).

Wrongly annotated NLRs in the Westar genome fell into 3 main categories: (i) predicted NLR-encoding genes absent in the annotation file; (ii) wrongly annotated NLR-encoding genes in the annotation file; and (iii) annotated NLR-encoding genes in the annotation file that contained 2 or more NLRs (Fig. 1B to D). For example, we found the gene model of the *Crr1a* allele, a known clubroot resistance gene originally found in resistant *B. rapa* cultivar “G004' (Hatakeyama et al. 2013), to be wrongly annotated in the Westar genome (Fig. 1C). To correctly annotate the remaining positions where RenSeq read and NLR-Annotator coverages were present, but gene annotations were either missing or partial, we wrote a custom script to extract such regions with 1 Kb flanking positions and with at least 50× RenSeq reads depth. AUGUSTUS (Hoff and Stanke 2013) was used to de novo predict genes in the extracted regions and the longest isoform was selected for further analysis. Using InterProScan with the Pfam database, we found 370 of those sequences to carry NLR-associated domains. Thus, by combining RenSeq mapping, the NLR-Annotator annotation, and de novo gene prediction of problematic regions using AUGUSTUS, we increased the number of correctly annotated NLR genes from 345 to 715 (Supplementary Tables S1 to S2). NLRtracker was used to classify the 715 amino acid sequences based on their domain composition, identifying 287 full NLRs and 428 partial NLRs in the Westar genome (Fig. 2A; Supplementary Table S1). Chromosomes C09 and A09 were found to have the most abundant NLRs, with 75 and 68, respectively (Fig. 2B; Supplementary Table S2). However, only 9 NLRs are present on chromosome A10 (Fig. 2B; Supplementary Table S2).

**Figure 2.**
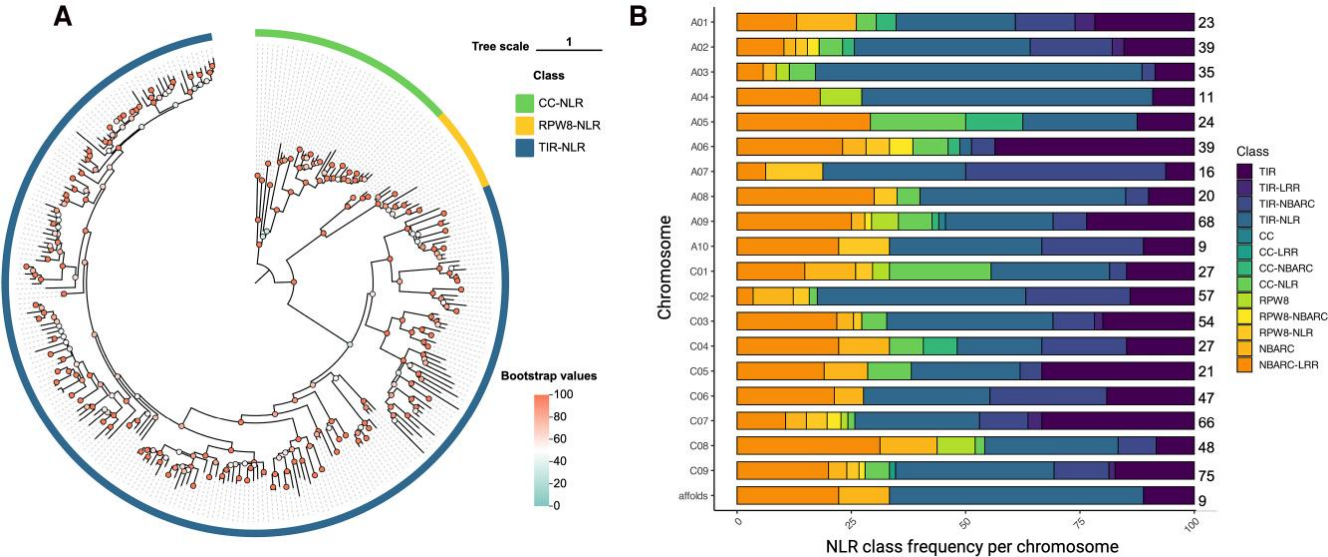
Phylogeny of Westar full NLRs and frequency of NLR classes in genomes A and C of Westar canola. **A)** Phylogenetic relationship among the 287 full NLRs in the Westar genome. The maximum likelihood phylogenetic tree was constructed based on NBARC domain sequences with 1000 bootstrap replicates, and JTT + F + R8 model was chosen was estimated to be the best model. The phylogenetic tree is visualized by tvBOT (Xie et al. 2023). Clades with 3 colors represent different types of full NLRs including CC-NLR, RPW8-NLR, and TIR-NLR, and the bootstrap value of each sublineage is shown by dot with different colors. **B)** Bar chart showing the percentage of different NLR classes in each chromosome and the scaffolds after reannotation of the Westar canola NLRome. The total number of NLRs on the 19 chromosomes and the scaffolds is also presented.

Among the full NLRs, there are 232 Toll/interleukin 1 receptor (TIR)-NLRs, 39 coiled-coil (CC)-NLRs, and 16 resistance to powdery mildew coiled-coil (RPW8)-NLRs, diversity that was confirmed through a maximum likelihood phylogenetic analysis using IQ-TREE2 based on the conserved NBARC domains (Fig. 2A; Supplementary Table S2 and File S3) (Minh et al. 2020). The C-terminal jelly roll/Ig-like domain (C-JID) domain, a domain found in TIR-NLRs, is essential for the recognition of pathogen effectors (Ma et al. 2020; Martin et al. 2020). We identified 199 NLRs containing the C-JID domain in the Westar genome (e-value < 1e-5), which represents 43.2% of the whole TIR-contained proteins (Supplementary Table S3). Out of the 428 partial NLRs, 138 sequences were found to only carry the TIR domain.

In addition to the canonical NLRs, we identified the integrated domains of NLRs (NLR-ID) in the NLRome of Westar canola by querying the Pfam database using InterProScan (e-value < 1e-5). Integrated domains often act as decoys, resembling crucial host protein components targeted by pathogen effectors, and play a key role in initiating the defense response upon effector recognition (Marchal et al. 2022). A total of 69 NLRs were found to carry integrated domains, and 49 different types of domains were identified (Supplementary Fig. S3 and Table S4). The galactose oxidase domain was the most prevalent in 8 NLRs, with 3 tandemly duplicated NLRs carrying multiple copies of the domain (Supplementary Fig. S3 and Table S4). Other known integrated domains, such as the heavy-metal associated domains, B3 DNA-binding domains, zinc-finger domains, and protein-kinase domains, were also present (Supplementary Fig. S3 and Table S4).

By providing a near-complete NLR repertory for *B. napus*, our study serves as a vital resource for the plant biotechnology community, fostering further research, and application in crop species. For example, we are now able to analyze synteny comparison of NLR-encoding genes between the sub-genomes A and C of *B. napus* (Supplementary Fig. S4), something that was not possible with the wrongly annotated Westar genome. Moreover, compared to other NLRome studies, the Westar NLRome provides the first complete open reading frame, start and stop codons, which can give more information to researchers and breeders (Supplementary File S4 and S5). These findings an important advancement in understanding canola genetics and offer practical applications for breeding programs and biotechnology aimed at improving disease resistance.

## Supplementary Material

kiae631_Supplementary_Data

## Data Availability

The raw data generated in this study is available in the Bioproject: PRJNA1137270, BioSample SAMN42564584, and SRA SRS22046306. The code used can be found in GitHub link: https://github.com/Edelab/RenSeq_Westar_NLRome. The rest of the data that supports the ﬁndings are available in the Supplementary material.

## Supplementary data

The following materials are available in the online version of this article.

**Supplementary Figure S1.** Expanded schematic representation of workflow followed in this study. Sequenced Illumina RenSeq reads were mapped to the Westar reference genome by BWA-MEM 2. By comparing the reference annotation file with the NLR-Annotator file, we identified regions—primarily within previously unannotated sections of the genome—that displayed high RenSeq read depth and contained NLR-Annotator predicted loci, indicating potential NLR loci in the Westar canola genome. The figure illustrates 3 possible scenarios where the correct annotation was missed and shows how these regions were accurately annotated using AUGUSTUS.

**Supplementary Figure S2.** Frequency of NLR classes in genomes A and C of Westar canola genome before re-annotation. Bar chart showing the percentage of different NLR classes in each chromosome and the scaffolds. The total number of NLRs on the 19 chromosomes and the scaffolds is also presented.

**Supplementary Figure S3.** Examples of integrated domains in the Westar canola NLRome. Forty-nine different types of integrated domains were found in the Westar canola NLRome. The lollipop chart presents the top 10 domains based on counts.

**Supplementary Figure S4.** Syntenic analysis of NLR coding genes between the A and C sub-genomes of Westar canola. Colored block represents NLR gene duplication pairs between A and C sub-genomes. Chromosomes are scaled based on the number of NLR genes.

**Supplementary Table S1.** Full list of NLRs and types of Westar genome.

**Supplementary Table S2.** NLR numbers by chromosome and types.

**Supplementary Table S3.** Full list of NLRs with C-JID domains generated by NLRtracker.

**Supplementary Table S4.** Full list of NLRs with integrated domains.

**Supplementary File 1.** Nucleotide sequences sent to Arbor Biosciences for baits design.

**Supplementary File 2.** The predicted NLR loci annotated by NLR-Annotator.

**Supplementary File 3.** The Maximum likelihood phylogenetic tree output file.

**Supplementary File 4.** Amino acid sequences of identified NLR proteins in this study.

**Supplementary File 5.** The full annotated NLR genes of Westar canola genome.

## Dive Curated Terms

The following phenotypic, genotypic, and functional terms are of significance to the work described in this paper:

AUGUSTUS Gramene: AUGUSTUS is a program that predicts genes in eukaryotic genomic sequences.
AUGUSTUS Araport: AUGUSTUS is a program that predicts genes in eukaryotic genomic sequences.
