# Supplementary figures and images for: Resistance gene enrichment sequencing refines the Brassica napus NLRome
Source: Plant Physiol. 2024 Nov 28;197(3):kiae631. doi: 10.1093/plphys/kiae631 (PMC11884775; doi:10.1093/plphys/kiae631)

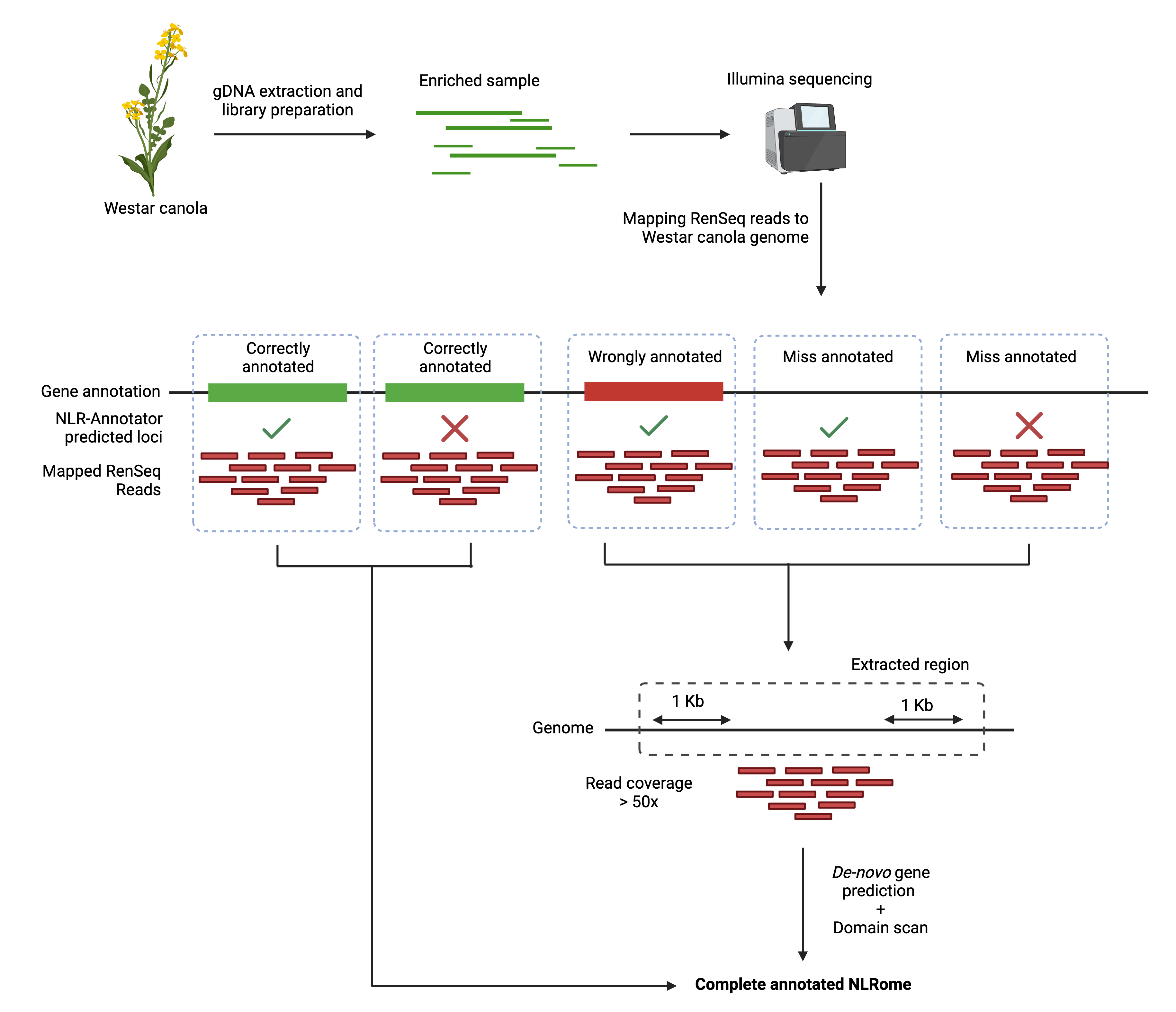

Supplement: kiae631_Supplementary_Data [file kiae631_supplementary_data.zip › PP2024LTR02190R1_Figure S1.png]

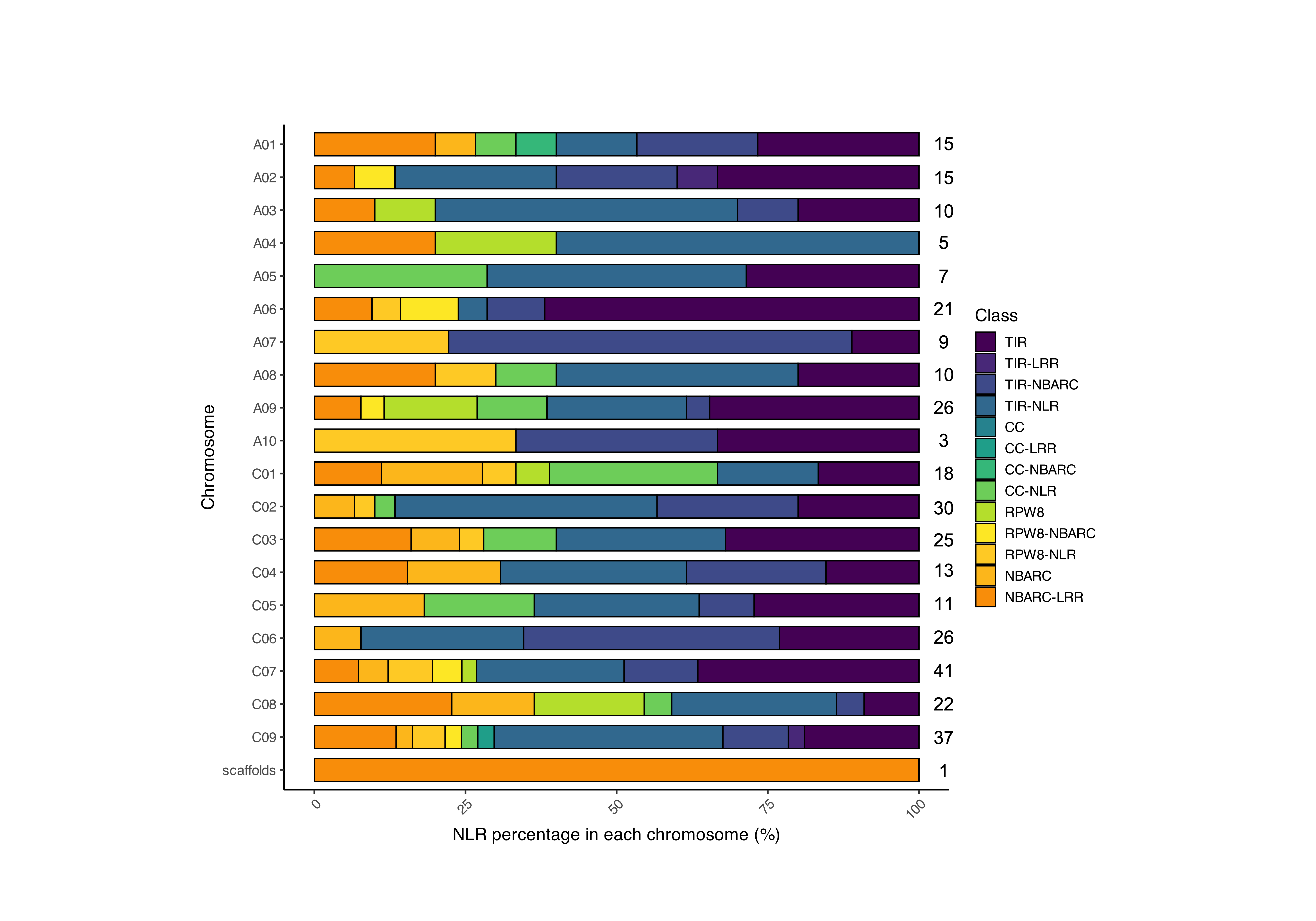

Supplement: kiae631_Supplementary_Data [file kiae631_supplementary_data.zip › PP2024LTR02190R1_Figure S2.png]

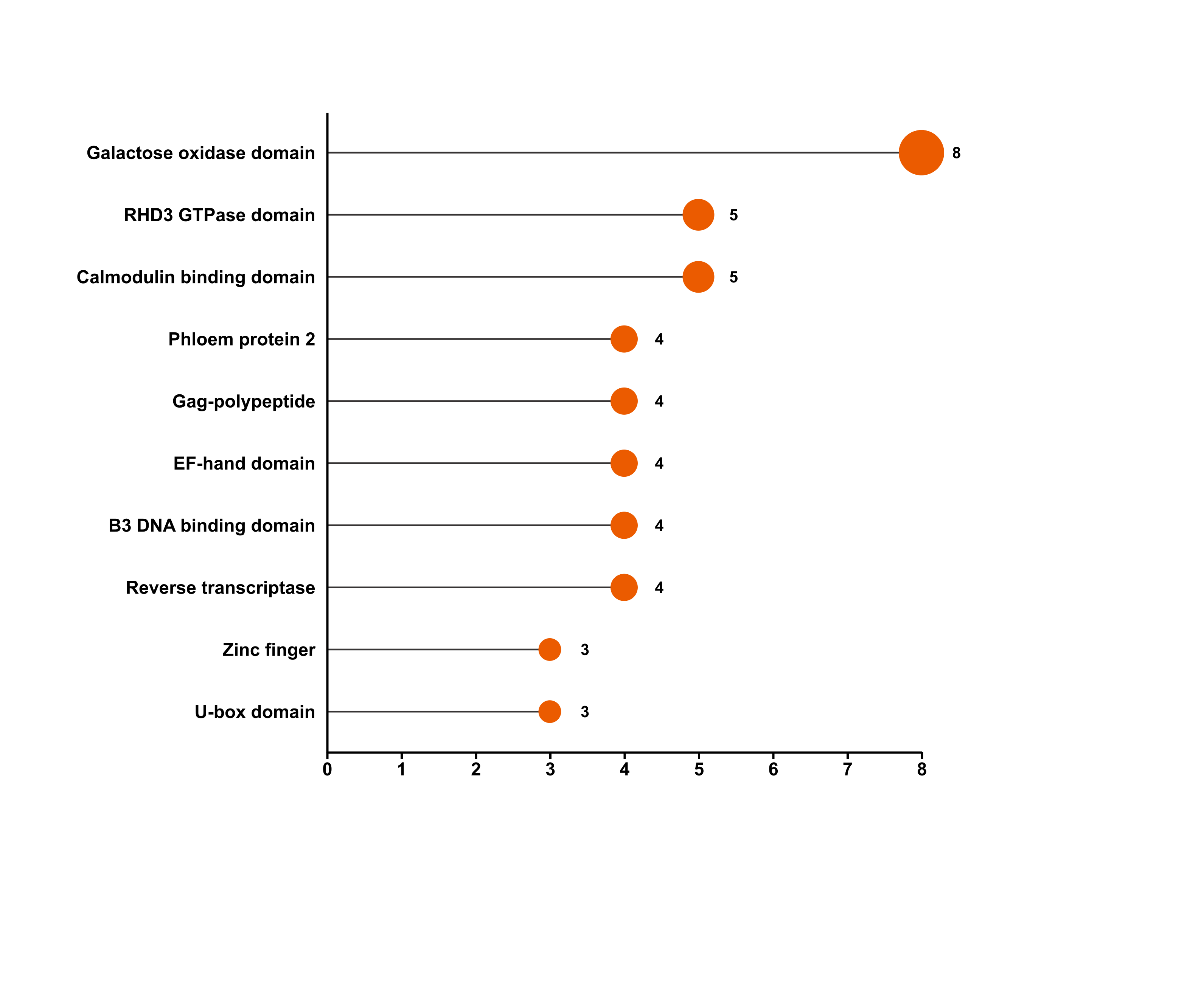

Supplement: kiae631_Supplementary_Data [file kiae631_supplementary_data.zip › PP2024LTR02190R1_Figure S3.png]

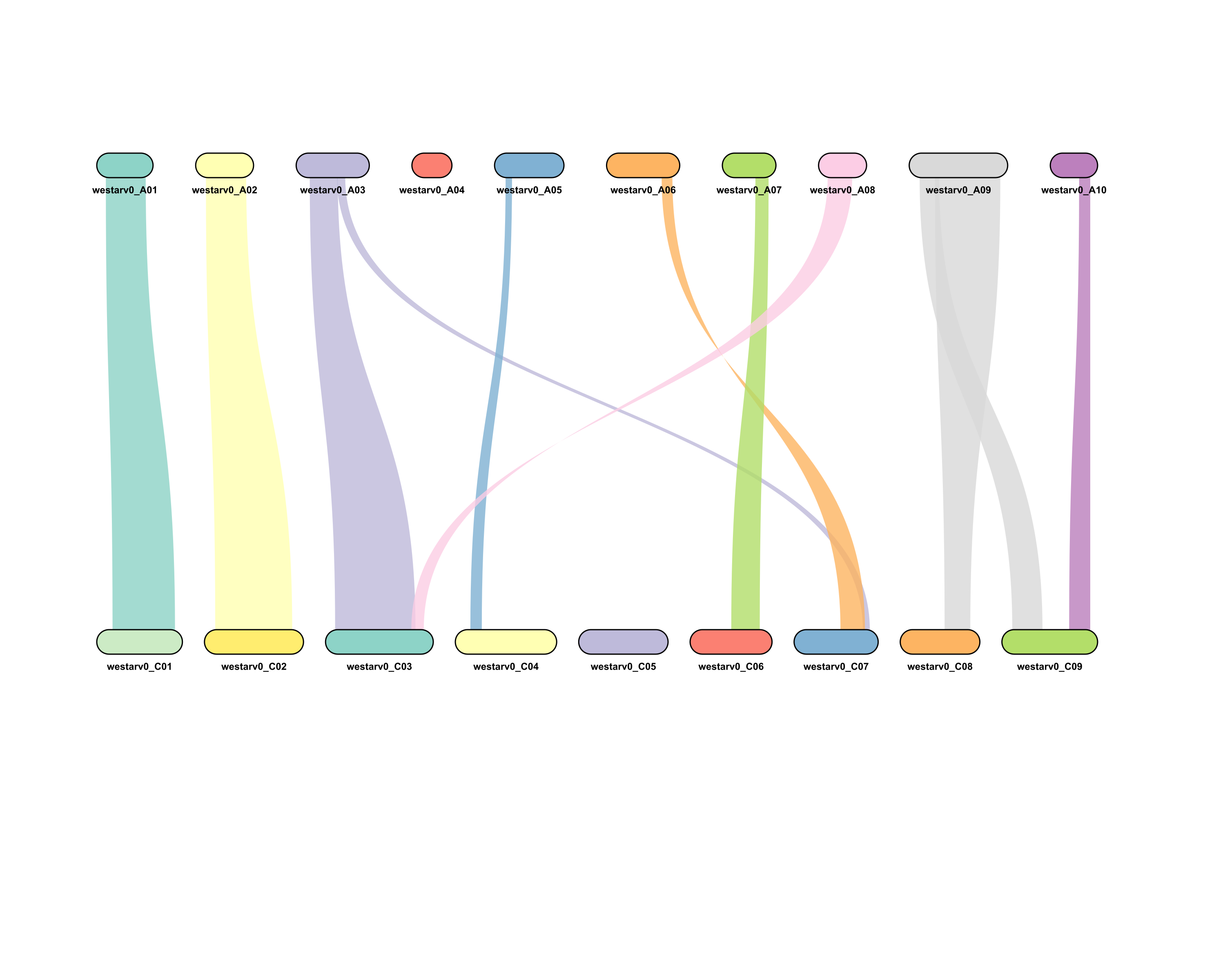

Supplement: kiae631_Supplementary_Data [file kiae631_supplementary_data.zip › PP2024LTR02190R1_Figure S4.png]
